# Lipidomic Analysis of the Outer Membrane Vesicles from Paired Polymyxin-Susceptible and -Resistant *Klebsiella pneumoniae* Clinical Isolates

**DOI:** 10.3390/ijms19082356

**Published:** 2018-08-10

**Authors:** Raad Jasim, Mei-Ling Han, Yan Zhu, Xiaohan Hu, Maytham H. Hussein, Yu-Wei Lin, Qi (Tony) Zhou, Charlie Yao Da Dong, Jian Li, Tony Velkov

**Affiliations:** 1 Drug Delivery, Disposition and Dynamics, Monash Institute of Pharmaceutical Sciences, Monash University, Parkville, Victoria 3052, Australia; raad.jasim@monash.edu (R.J.); charlie.dong@monash.edu (C.Y.D.D.); 2 Monash Biomedicine Discovery Institute, Immunity and Infection Program and Department of Microbiology, Monash University, VIC 3800, Australia; meiling.han@monash.edu (M.-L.H.); yan.zhu@monash.edu (Y.Z.); yu-wei.lin@monash.edu (Y.-W.L.); 3 Department of Pharmacology and Therapeutics, University of Melbourne, Parkville, Victoria 3010, Australia; xiaohanh2@student.unimelb.edu.au (X.H.); maytham.hussein@unimelb.edu.au (M.H.H.); 4 Department of Industrial and Physical Pharmacy, College of Pharmacy, Purdue University, 575 Stadium Mall Drive, West Lafayette, IN 47907, USA; tonyzhou@purdue.edu

**Keywords:** outer membrane vesicles, lipidomics, Gram-negative, polymyxin, extremely drug resistant

## Abstract

Gram-negative bacteria produce outer membrane vesicles (OMVs) as delivery vehicles for nefarious bacterial cargo such as virulence factors, which are antibiotic resistance determinants. This study aimed to investigate the impact of polymyxin B treatment on the OMV lipidome from paired polymyxin-susceptible and -resistant *Klebsiella pneumoniae* isolates. *K. pneumoniae* ATCC 700721 was employed as a reference strain in addition to two clinical strains, *K. pneumoniae* FADDI-KP069 and *K. pneumoniae* BM3. Polymyxin B treatment of the polymyxin-susceptible strains resulted in a marked reduction in the glycerophospholipid, fatty acid, lysoglycerophosphate and sphingolipid content of their OMVs. Conversely, the polymyxin-resistant strains expressed OMVs richer in all of these lipid species, both intrinsically and increasingly under polymyxin treatment. The average diameter of the OMVs derived from the *K. pneumoniae* ATCC 700721 polymyxin-susceptible isolate, measured by dynamic light scattering measurements, was ~90.6 nm, whereas the average diameter of the OMVs isolated from the paired polymyxin-resistant isolate was ~141 nm. Polymyxin B treatment (2 mg/L) of the *K. pneumoniae* ATCC 700721 cells resulted in the production of OMVs with a larger average particle size in both the susceptible (average diameter ~124 nm) and resistant (average diameter ~154 nm) strains. In light of the above, we hypothesize that outer membrane remodelling associated with polymyxin resistance in *K. pneumoniae* may involve fortifying the membrane structure with increased glycerophospholipids, fatty acids, lysoglycerophosphates and sphingolipids. Putatively, these changes serve to make the outer membrane and OMVs more impervious to polymyxin attack.

## 1. Introduction

Over the last decade, extremely drug-resistant (XDR) *Klebsiella pneumoniae* has emerged as one of the most deadly Gram-negative ‘superbugs’ [1,2,3]. *K. pneumoniae* is responsible for numerous lethal nosocomial outbreaks [4]; more worryingly, the mortality of nosocomial *K. pneumoniae* infections can be up to 50% [5]. Carbapenem resistance in *K. pneumoniae* mediated by carbapenemase was firstly reported in 1996 in New York City and has spread to most global centres [5,6]. In 2008, *bla_NDM-1_*, which encodes the class B New Delhi Metallo-β-lactamase-1 (NDM-1) that inactivates carbapenems, was first detected in a Swedish patient who had contracted an infection in India [7]. Polymyxins (i.e., colistin and polymyxin B) are increasingly used as the last-line therapy against XDR *K. pneumoniae* [8]. Indeed, considerable in vitro activity against *K. pneumoniae* strains has been demonstrated [9]; 98.2% of general clinical strains of *K. pneumoniae* are susceptible to polymyxin B and colistin [10,11,12,13,14,15]. Ominously, XDR strains that are resistant to polymyxins have recently emerged [16,17], which highlights the need for a greater appreciation of the mechanism(s) of polymyxin resistance in *K. pneumoniae* to assist targeted drug discovery strategies.

The Gram-negative outer membrane (OM) constitutes a formidable barrier limiting the permeability of various noxious substances such as antimicrobial drugs [18,19]. This complex asymmetrical structure comprises an inner phospholipid leaflet, as well as an outer leaflet that predominantly contains lipopolysaccharide (LPS), proteins and phospholipids. Additionally, *K. pneumoniae* commonly expresses a capsular polysaccharide that coats the OM, the expression levels of which have been related to polymyxin susceptibility [20,21,22,23]. The antimicrobial action of polymyxins is mediated through a direct and very specific interaction with the lipid A component of the LPS, which leads to a disruption of the OM barrier [8]. The cationic l-α,γ-diaminobutyric acid residues of the polymyxin molecule produce an electrostatic attraction to the negatively charged lipid A phosphate groups, displacing the divalent cations (Mg^2+^ and Ca^2+^) [8]. The displacement leads to the disorganization of the LPS leaflet, enabling the insertion of the hydrophobic tail and the hydrophobic side chains of amino acids 6 and 7 of the polymyxin molecule into the OM [24]. Polymyxin resistance in *K. pneumoniae* primarily involves the multi-tier upregulation of capsular polysaccharide expression, and the systems required for the modification of lipid A with 4-amino-4-deoxy-l-arabinose and palmitoyl addition [20,23,25,26,27,28,29,30,31,32]. In *K. pneumoniae* the expression of 4-amino-4-deoxy-l-arabinose modifications to the lipid A phosphates is under control of the two component regulatory systems [PhoPQ–PmrD]–PmrAB that are activated in response to low pH, low magnesium, high iron and in response to cationic antimicrobial peptides [23]. More specifically, PhoP–PhoQ regulates the magnesium regulon, which activates polymyxin resistance under low magnesium conditions. This PhoP–PhoQ system is connected by the small basic protein PmrD. PhoP regulates the activation of PmrD, which can then bind to PmrA and prolong its phosphorylation state, eventually activating the expression of the PmrA–PrmB system to promote lipid A modifications that confer polymyxin resistance. The under-acylation of lipid A increases the polymyxin susceptibility of *K. pneumoniae*, which highlights that the decoration of lipid A with additional fatty acyl chains is important for polymyxin resistance [33,34].

Gram-negative bacteria naturally shed their OM via outer membrane vesicles (OMVs), which are spherical bilayer structures of approximately 20–200 nm in diameter [35]. OMVs are believed to serve as delivery vehicles for nefarious bacterial cargo such as virulence factors, antibiotic resistance determinants, toxins and factors that modulate the host immune response to facilitate pathogen evasion [35,36,37,38,39]. This underscores the need to understand the compositional differences between OMVs of MDR *K. pneumoniae* clinical isolates and how this relates to their pathogenicity. In the present study, we aimed to perform a comparative analysis of the lipidome of OMVs isolated from of polymyxin-susceptible and -resistant *K. pneumoniae* clinical isolates and to identify key lipid species that are selectively packaged from the OM into the OMV sub-lipidome of the resistant isolates. The obtained data sheds new light on the OMV lipidomes associated with high-level polymyxin resistance in the problematic Gram-negative pathogen *K. pneumoniae*.

## 2. Results and Discussion

### 2.1. Lipidomics Analysis of OMVs from Polymyxin-Susceptible and -Resistant K. pneumoniae Isolates

The OMV lipidome from paired polymyxin-susceptible and -resistant strains from two clinical isolates (*K. pneumoniae* BM3 and FADDI-KP069) and a laboratory type strain (*K. pneumoniae* ATCC 700721) were characterised following lipid extraction using LC-MS analysis. Compositional analysis revealed that the OMV lipid composition of all the *K. pneumoniae* strains mostly consisted of glycerophospholipids (~35%), fatty acids (~33%) and sphingolipids (~20%). Similarly, across all three strains the OMV minor lipid components consisted of lipids from the following classes, glycerolipids (~4%), sterol lipids (~3%) and prenol lipids (~4%).

**Figure 1:**
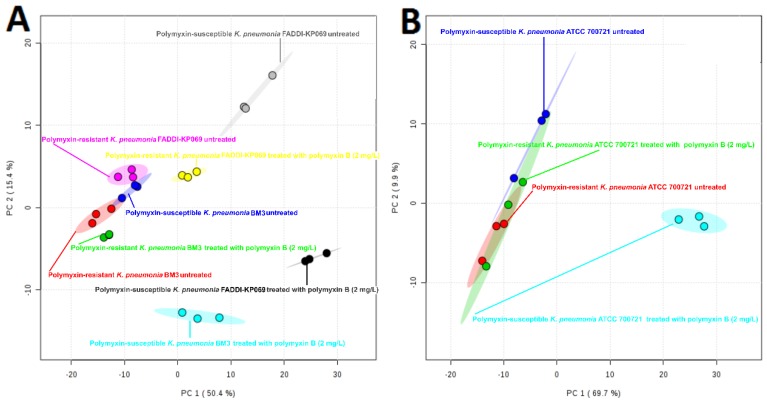
Principal component analysis (PCA) score plot for OMVs isolated from polymyxin-susceptible and -resistant *K. pneumoniae* isolates. (**A**) PCA score plot for the two clinical isolates. Polymyxin-resistant *K. pneumoniae* BM3 untreated (red); polymyxin-resistant *K. pneumoniae* BM3 treated with polymyxin B (2 mg/L) (green); polymyxin-susceptible *K. pneumoniae* BM3 untreated (blue); polymyxin-susceptible *K. pneumoniae* BM3 treated with polymyxin B (2 mg/L) (cyan); polymyxin-resistant *K. pneumoniae* FADDI-KP069 untreated (purple); polymyxin-resistant *K. pneumoniae* FADDI-KP069 treated with polymyxin B (2 mg/L) (yellow); polymyxin-susceptible *K. pneumoniae* FADDI-KP069 untreated (grey); polymyxin-susceptible *K. pneumoniae* FADDI-KP069 treated with polymyxin B (2 mg/L) (black). (**B**) PCA score plot for the paired *K. pneumoniae* ATCC 700721 laboratory type isolates. Polymyxin-resistant *K. pneumoniae* ATCC 700721 untreated (red); polymyxin-resistant *K. pneumoniae* ATCC 700721 treated with polymyxin B (2 mg/L) (green); polymyxin-susceptible *K. pneumoniae* ATCC 700721 untreated (blue); polymyxin-susceptible *K. pneumoniae* ATCC 700721 treated with polymyxin B (2 mg/L) (cyan). Each data point represents three biological replicates.

**Figure 2:**
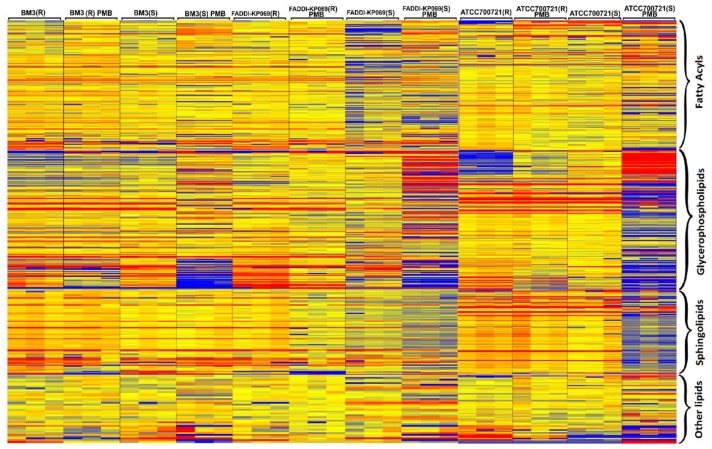
The heat map illustrates the relative peak intensity of lipids within each class in the OMVs of the paired polymyxin-susceptible and -resistant *K. pneumoniae* isolates. (R) = polymyxin-resistant; (S) = Polymyxin-susceptible. Colours indicate relative abundance of lipidomes based on the relative peak intensity (red = high, yellow = no change, blue = undetectable).

**Figure 3:**
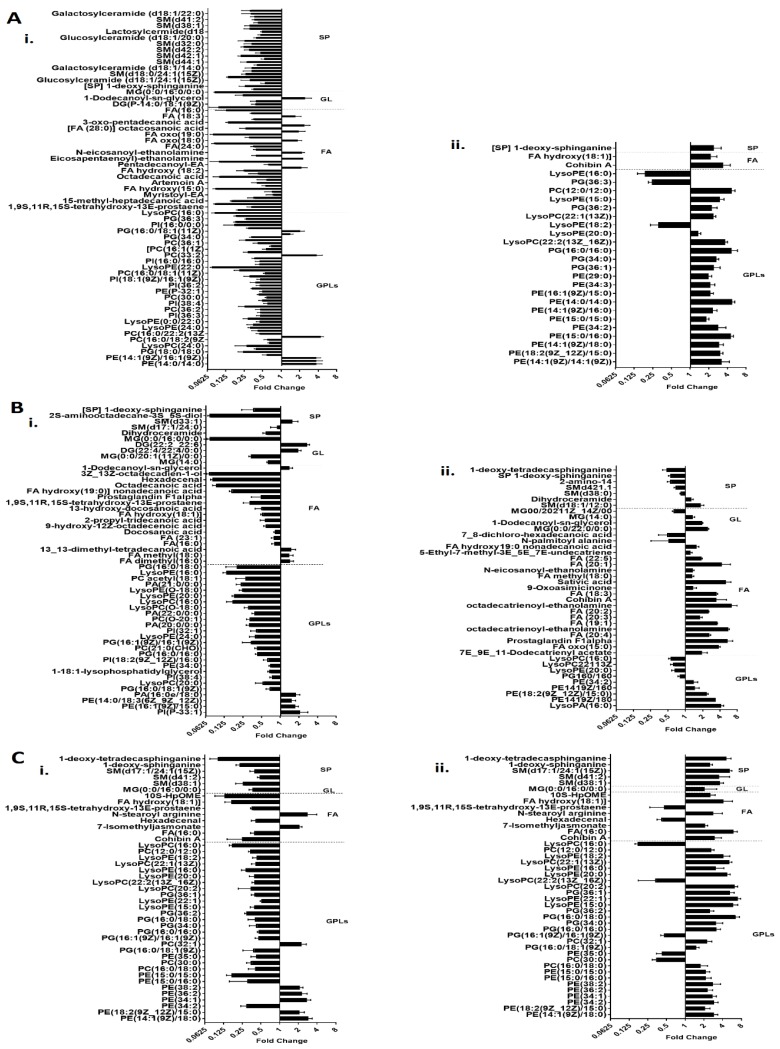
Lipidomic perturbations of OMVs isolated from polymyxin-susceptible and -resistant *K. pneumoniae* isolates. Fold-change of lipids relative to the untreated control cells, in OMVs of the polymyxin-susceptible (**i**) and -resistant (**ii**) strains of paired *K. pneumoniae* isolates in response to polymyxin B treatment (2 mg/L). (**A**) *K. pneumoniae* ATCC 700721. (**B**) *K. pneumoniae* BM3 and (**C**) *K. pneumoniae* FADDI-KP069. GPLs = glycerophospholipids; FA = fatty acids; GL = glycerolipids; SP = sphingolipids.

**Figure 4:**
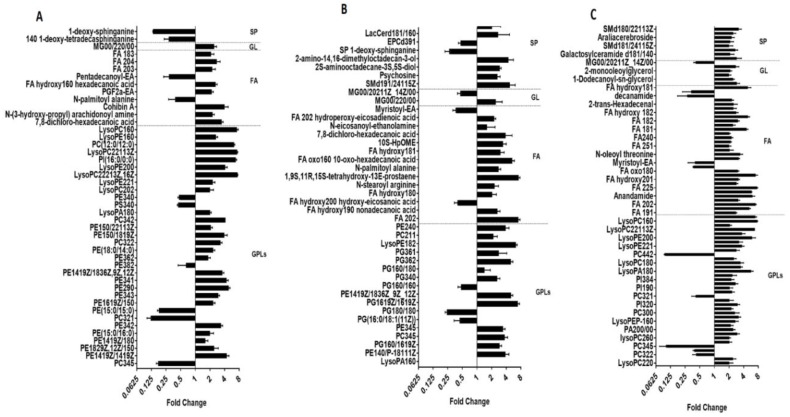
Major differences in the lipid abundance between the OMVs of paired polymyxin-susceptible and -resistant *K. pneumoniae* isolates. The differences are expressed as the fold-change in the OMV lipids of the paired susceptible vs. resistant *K. pneumoniae* isolates. All cultures were grown in the absence of polymyxins. (**A**) *K. pneumoniae* ATCC 700721. (**B**) *K. pneumoniae* BM3 and (**C**) *K. pneumoniae* FADDI-KP069. GPLs = glycerophospholipids; FA = fatty acids; GL = glycerolipids; SP = sphingolipids.

Principle component analysis (PCA) score plots and the heat map revealed significant global lipidomic differences between the OMVs of the polymyxin-susceptible and -resistant *K. pneumoniae* strains (Figure 1 and Figure 2). Notably, following treatment with a clinically relevant concentration of polymyxin B (2 mg/L) we observed marked global lipidome perturbations in the OMVs of the polymyxin-susceptible *K. pneumoniae* strains; whereas the OMVs of the resistant strains showed moderate global lipidome perturbations in response to polymyxin B treatment of the cells. For univariate analyses, all of the putatively identified lipids were further analysed to reveal those showing at least 2-fold differences (*p* < 0.05, FDR < 0.05, one-way ANOVA test) in relative abundance (Figure 3). The cluster algorithm and fold-change analysis highlighted that, compared to the untreated controls, polymyxin B treatment (2 mg/L) of the polymyxin-susceptible *K. pneumoniae* ATCC 700721 significantly reduced the phosphatidylcholine, phosphatidylethanolamine and 1-acyl-glycerophosphocholine content of its OMVs. Additionally, the sphingolipids namely, sphingosine, *n*-acyl-sphingosine (ceramide), *n*-acyl-sphinganine(dihydro-ceramide), sphingomyelin, glucosyl-ceramide and lactosyl-ceramide were significantly reduced following polymyxin B treatment (Figure 3Ai). Moreover, certain saturated fatty acids (e.g., hexadecanoic acid and octadecanoic acid), and polyunsaturated fatty acids (α-linolenic acid and arachidonic acid) were also reduced in the OMVs of the polymyxin B treated susceptible isolate. Polymyxin B treatment of the its paired polymyxin-resistant *K. pneumoniae* ATCC 700721 laboratory isolate significantly increased the content of lysoglycerophosphates, phosphatidylcholines and phosphatidylethanolamines in its OMVs (Figure 3Aii). Similarly, to the polymyxin-susceptible ATCC 700721 isolate, most of the glycerophospholipid and fatty acid content of the OMVs isolated from the polymyxin B treated polymyxin-susceptible clinical isolates (*K. pneumoniae* BM3 and FADDI-KP069) were significantly reduced compared to untreated controls (Figure 3Bi,Ci). In particular, glycerophospholipids (e.g., phosphatidylethanolamines, phosphatidylcholines, lysophosphatidylcholines, and lysoglycerophosphates) were remarkably reduced in response to polymyxin B treatment. In addition, fatty acids (e.g., docosanoic acid, octadecenoic acid and hexadecanoic acid); and sphingolipids (mainly dihydro-ceramides) were also significantly reduced in response to polymyxin B be treatment. In contrast, the majority of glycerophospholipids, fatty acids and sphingolipids content of OMVs isolated from their paired polymyxin-resistant *K. pneumoniae* BM3 a FADDI-KP069 isolates were significantly increased in response to polymyxin B treatment (Figure 3Bii,Cii). Notably, all of the polymyxin B-resistant strains secreted OMVs significantly are richer in glycerophospholipids, fatty acids, lysoglycerophosphates and sphingolipids compared to polymyxin B-susceptible isolates even when grown in the absence of polymyxin B (Figure 4). Glycerophospholipids, fatty acids, glycerolipids and sphingolipids play a crucial role in maintain outer membrane integrity, bacterial survival and pathogenesis [40]. Phospholipids (including glycerophospholipids) are essential components of bacterial membranes and they are responsible for maintaining membrane integrity and the selective permeability of the outer membrane [41]; they contribute to cationic peptide resistance, protect bacteria from osmotic stress and regulate flagellum-mediated motility [42]. In addition, sphingolipids are involved in maintaining normal bacterial growth and membrane integrity; and trigger bacterial pathogenesis via induction of the host immune system [43]. 

### 2.2. Transmission Electron Microscopy Imaging and Dynamic Light Scattering Size Estimation of K. pneumoniae OMVs 

**Figure 5:**
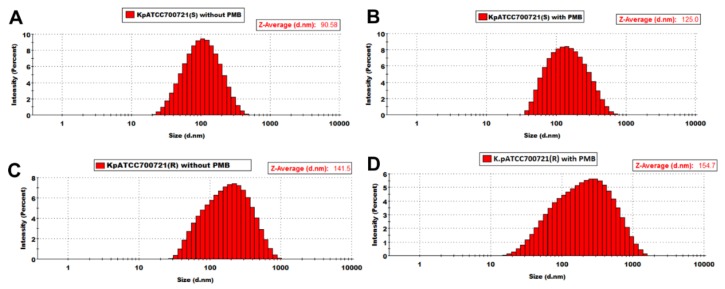
Size distribution measured by dynamic light scattering of OMVs isolated from paired polymyxin-susceptible and -resistant strains of *K. pneumoniae* ATCC 700721. OMVs isolated from the polymyxin-susceptible *K. pneumoniae* ATCC 700721 (**A**) without polymyxin B treatment and (**B**) with polymyxin B (2 mg/L) treatment. OMVs isolated from the polymyxin-resistant *K. pneumoniae* ATCC 700721 (**C**) without polymyxin B treatment and (**D**) with polymyxin B (2 mg/L) treatment.

**Figure 6:**
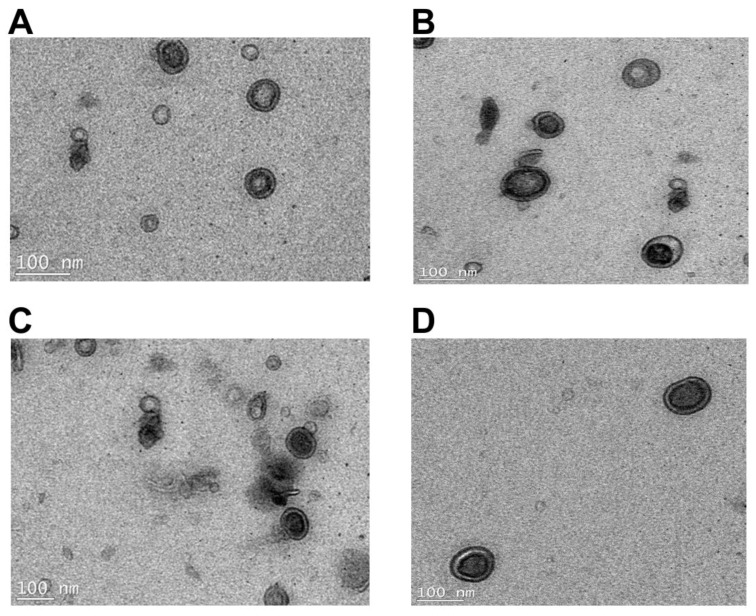
Transmission electron microscopy images of OMVs isolated from paired polymyxin-susceptible and -resistant strains of *K. pneumoniae* ATCC 700721. (**A**) OMVs from untreated *K. pneumoniae* ATCC 700721 (susceptible). (**B**) OMVs from polymyxin B (2 mg/L) treated *K. pneumoniae* ATCC 700721 (susceptible). (**C**) OMVs from untreated *K. pneumoniae* ATCC 700721 (resistant). (**D**) OMVs from polymyxin B (2 mg/L) treated *K. pneumoniae* ATCC 700721 (resistant).

Dynamic light-scattering (DLS) analysis revealed that the average hydrodynamic radius of the OMVs derived from the *K. pneumoniae* ATCC 700721 polymyxin-susceptible isolate is ~90.6 nm; the profile was symmetrical and the OMV scatter ranged from ~30–500 nm (Figure 5A). The average hydrodynamic radius of the OMVs isolated from the paired *K. pneumoniae* ATCC 700721 polymyxin-resistant isolate was ~141 nm and the OMV scatter ranged from ~30 to 1000 nm (Figure 5C), which indicates that the resistant isolae sheds larger OMVs than the susceptible one. Polymyxin B treatment (2 mg/L) of the *K. pneumoniae* cells resulted in the production of OMVs with slightly larger average particle size in both the susceptible (average diameter ~124 nm, OMV scatter ~30–900 nm; Figure 5B) and resistant (average hydrodynamic radius ~154 nm, OMV scatter ~30–1500 nm; Figure 5D) strains. Notably, the OMV scatter profile in the resistant strain is asymmetrical, with and without polymyxin B treatment. In line with the DLS data [44,45], transmission electron microscopy imaging of *K. pneumoniae* OMVs revealed a similar size distribution wherein the polymyxin-resistant *K. pneumoniae* ATCC 700721 strain produced larger OMVs than the susceptible strain (Figure 6). Moreover, the OMVs isolated from the polymyxin-resistant isolate stained darker with the TEM contrast reagent uranyl acetate, which enhances the contrast by interaction with lipids; in line with the lipidomics findings, this would suggest that the OMVs of the resistant strains contain more lipids. Similarly, in *Salmonella enterica*, LPS remodelling in the outer membrane in response to polymyxins or other environmental PhoP/Q–PmrA/B activating conditions, has been shown to stimulate the biogenesis of larger-diameter OMVs [36,37,38,39].

## 3. Materials and Methods

### 3.1. Materials

Polymyxin B was supplied by Betapharma (Shanghai, China). All chemicals were purchased from Sigma-Aldrich (Melbourne, VIC, Australia) at the highest research grade; ultrapure water was from Fluka (Castle Hill, New South Wales, Australia). Stock solutions of polymyxin B (10 mg/L) were freshly prepared in ultrapure water and filtered through 0.22 µm syringe filters (Sartorius, Melbourne, Victoria, Australia). 

### 3.2. Bacterial Isolates and Growth Conditions

All bacterial strains used in this study are described in Table S1. Resistance to polymyxin B was defined as MICs of ≥8 mg/L [46]. A total of six different *K. pneumoniae* isolates were studied: The clinical isolates *K. pneumoniae* FADDI-KP069 (polymyxin-susceptible strain polymyxin B MIC = 0.5 mg/L; polymyxin-resistant strain polymyxin B MIC > 32 mg/L; Both positive for ESBL and KPC carbapenemase) and *K. pneumoniae* BM3 (polymyxin-susceptible strain polymyxin B MIC = 0.5 mg/L; polymyxin-resistant strain polymyxin B MIC ≥ 32 mg/L; Both positive for NDM, CTX-M, SHV, TEM, AAC-6’-1B); and a reference strain *K. pneumoniae* ATCC 700721 (polymyxin-susceptible strain polymyxin B MIC = 0.5 mg/L; polymyxin-resistant strain polymyxin B MIC > 32 mg/L). The antibiograms of the two clinical isolates are documented in Table S1. All bacteria were stored at −80 °C in tryptone soya broth (TSB, Oxoid, Melbourne, Australia). Prior to experiments, parent strains were subcultured onto nutrient agar plates (Medium Preparation Unit, University of Melbourne, Victoria, Australia). Overnight broth cultures were subsequently grown in 5 mL of cation-adjusted Mueller–Hinton broth (CaMHB, Oxoid, West Heidelberg, Victoria, Australia), from which a 1 in 100 dilution was performed in fresh broth to prepare mid-logarithmic cultures according to the optical density at 500 nm (OD_500nm_ = 0.4 to 0.6). All broth cultures were incubated at 37 °C in a shaking water bath (180 rpm). 

### 3.3. Minimum Inhibitory Concentration (MIC) Microbiological Assay

MICs were performed according to the Clinical and Laboratory Standards Institute (CLSI) guidelines [47]. MICs were determined for all isolates in three replicates on separate days using broth microdilution method in cation-adjusted Mueller–Hinton broth (CAMHB) in 96-well polypropylene microtitre plates. Wells were inoculated with 100 µL of bacterial suspension prepared in CaMHB (containing 10^6^ colony-forming units (cfu) per mL) and 100 µL of CaMHB containing increasing concentrations of polymyxin B (0.25–256 mg/L). The MICs were defined as the lowest concentration at which visible growth was inhibited following 18 h incubation at 37 °C. Cell viability was determined by sampling wells at polymyxin B concentrations greater than the MIC. These samples were diluted in normal saline and spread plated onto nutrient agar. After incubation at 37 °C for 20 h, viable colonies were counted on these plates. The limit of detection was 10 cfu/mL. 

### 3.4. Isolation of Outer Membrane Vesicles (OMVs)

Mid-logarithmic cultures (6 L) of each isolate were grown at 37 °C with shaking (1800 rpm) and cell-free supernatants were collected through centrifugation (15 min at 10,000× *g*, 4 °C). Where indicated, polymyxin B was added to the culture volume at a final concentration of 2 mg/L. The OMV containing supernatants were filtered through 0.22-μm membrane (Sigma-Aldrich) to remove any remaining cell debris, then concentrated through a tangential filtration concentrator unit (Pall Life Science, Ann Arbor, MI, USA) and collected using 100 kDa Pellicon filtration cassettes (Millipore, Melbourne, Australia). Also, a portion of the supernatant was plated for growth on agar plates overnight at 37 °C to make sure that the supernatant is free of bacterial cells. OMVs in the cell-free supernatants were then pelleted down by ultracentrifugation at 150,000× *g* for 2 h at 4 °C in a Beckman Ultracentrifuge (SW28 rotor). Purified OMVs were concentrated re-suspended in 1 mL sterile PBS and the concentration was determined by Bio-Rad (Gladesville, NSW, Australia) protein assay. 

### 3.5. Lipidomics Analysis

OMV lipids were extracted with the single-phase Bligh–Dyer method (CHCl_3_/MeOH/H_2_O, 1:3:1, *v*/*v*) [48]. For further analysis, samples were reconstituted in 100 µL of CHCl_3_ and 200 µL of MeOH, centrifuged at 14,000× *g* for 10 min at 4 °C to obtain particle-free supernatants. LC-MS for lipidomic analysis was conducted on a Dionex U3000 high-performance liquid chromatography system (HPLC) in tandem with a Q-Exactive Orbitrap mass spectrometer (Thermo Fisher, Melbourne, Australia) in both positive and negative mode with a resolution at 35,000. The mass scanning range was from 167 to 2000 *m*/*z*. The electrospray voltage was set as 3.50 kV and nitrogen was used as collision gas. The Ascentis Express C_8_ column (5 cm × 2.1 mm, 2.7 µm, Sigma-Aldrich, 53831-U) was maintained at 40 °C, and the samples were controlled at 4 °C. The flow rate was 0.2 mL/min at first 24 min, but increased to 0.5 mL/min from 25 min to 30 min. The multi-step gradient started from 100% to 80% mobile phase A over the first 1.5 min, then to 72% mobile phase A at 7 min, over the next 1 min, the gradient changed to 65% mobile phase A, from 8 min to 24 min, the gradient reached a final composition of 35% mobile phase A and 65% mobile phase B. This was followed by a washing step from 65% to 100% mobile phase B over the next 1 min, and maintained for 2 min. A 2-min re-equilibration of the column with 100% A was performed between injections. Untargeted lipidomic analyses were performed through mzMatch [49]; and IDEOM [50] (http://mzmatch.sourceforge.net/ideom.php). Raw LC-MS data files were converted to mzXML format through a proteowizard tool, Msconvert. Automated chromatography peaks were picked by XCMS [51], and then converted to peakML files, which were combined and filtered by mzMatch based on the intensity (1000), reproducibility (RSD for all replicates < 0.8), and peak shape (codadw > 0.8). The mzMatch program was used for retrieving intensities for missing peaks and the annotation of related peaks. Unmatched peaks and noises were rejected through IDEOM. The database used in IDEOM included KEGG, MetaCyc and Lipidmaps [52]. Univariate statistics analysis was performed using a Welch’s T-test (*p* < 0.05), while multivariate analysis was conducted using the metabolomics R package. 

### 3.6. Transmission Electron Microscopy (TEM)

Carbon-coated Formvar copper grids were placed on a drop of OMV suspension (1 mg/mL protein) for 5 min then washed three times with PBS and fixed in 1% glutaraldehyde for 4 min. Grids were then washed three times with PBS, two times with Milli-Q water and stained for 20 s with 4% uranyl acetate. Grids were finally washed with Milli-Q water and incubated on ice for 10 min in methyl–cellulose with 4% uranyl acetate (9:1). Grids were then air-dried and viewed with a Tecnai Spirit (T12) transmission electron microscope, and the images were acquired using TIA software (FEI, Melbourne, Australia). 

### 3.7. Dynamic Light Scattering

The particle size of the OMVs was measured using dynamic light scattering (DLS). OMVs were diluted with PBS to a protein concentration of 0.05 mg/L and the scatter was recorded using a Zetasizer NanoS (Malvern, PA, USA) at 173° with a laser of wavelength 632 nm. Data were analysed with Zetasizer Software (V7.11, Malvern, UK) to obtain the average hydrodynamic radius.

## 4. Conclusions

In this study, we show that polymyxin B treatment of the susceptible *K. pneumoniae* strains significantly reduced the glycerophospholipid, fatty acid, lysoglycerophosphate and sphingolipid content of their OMVs, compare to the untreated control. On the other hand, in the OMVs of their paired polymyxin-resistant strains these lipids were increased both intrinsically and in response to polymyxin B treatment. In view of these findings, it is reasonable to hypothesize that the outer membrane remodelling associated with polymyxin-resistance in *K. pneumoniae* entails fortifying the membrane with increased glycerophospholipids, fatty acids, lysoglycerophosphates and sphingolipids, which are lipids to which polymyxins cannot avidly bind. It is important to mention that polymyxins primarily target the lipid A in the Gram-negative outer membrane—hence their narrow spectrum of activity against Gram-negative bacteria that do not express LPS. These outer membrane changes may be accompanied by the modification of the lipid A with cationic moieties and/or a reduction in the lipid A content, which, together with the increased content of the aforementioned lipids, serve to make the *K. pneumoniae* outer membrane and OMVs more impervious to polymyxin attack. 

## Supplementary Materials

Supplementary materials can be found at: http://www.mdpi.com/1422-0067/19/8/2356/s1.
