# Supplementary material for: Lipidomic Analysis of the Outer Membrane Vesicles from Paired Polymyxin-Susceptible and -Resistant Klebsiella pneumoniae Clinical Isolates
Source: Int J Mol Sci. 2018 Aug 10;19(8):2356. doi: 10.3390/ijms19082356 (PMC6121281; doi:10.3390/ijms19082356)
Supplement: Supplementary file 1 [file ijms-19-02356-s001.zip › ijms-342629-SI.pdf]

International Journal of *Molecular Sciences*

MDPI

Article

Lipidomic Analysis of the Outer Membrane Vesicles from Paired Polymyxin-Susceptible and -Resistant *Klebsiella pneumoniae* Clinical Isolates

Raad Jasim [1], Mei-Ling Han [2], Yan Zhu [2], Xiaohan Hu [3], Maytham H. Hussein [3], Yu-Wei Lin [2], Qi (Tony) Zhou [4], Charlie Yao Da Dong [1], Jian Li [2,*] and Tony Velkov[3,*]

1 Drug Delivery, Disposition and Dynamics, Monash Institute of Pharmaceutical Sciences, Monash University, Parkville, 3052 Victoria, Australia; raad.jasim@monash.edu (R.J.); charlie.dong@monash.edu (C.Y.D.D)
2 Monash Biomedicine Discovery Institute, Immunity and Infection Program and Department of Microbiology, Monash University, 3800 VIC, Australia; meiling.han@monash.edu (M.H); yan.zhu@monash.edu (Y.Z); Jian.Li@monash.edu (J.L)
3 Department of Pharmacology and Therapeutics, University of Melbourne, Parkville, 3010 Victoria, Australia; xiaohanh2@student.unimelb.edu.au (X.H); maytham.hussein@unimelb.edu.au (M.H.H); tony.velkov@monash.edu (T.V.)
4 Department of Industrial and Physical Pharmacy, College of Pharmacy, Purdue University, 575 Stadium Mall Drive, West Lafayette, 47907 IN, USA; tonyzhou@purdue.edu
* Correspondence: colistin.polymyxin@gmail.com (J.L); Tony.Velkov@unimelb.edu.au (T.V)

Received: 29 July 2018; Accepted: 7 Aug 2018; Published: date

Supplementary Materials:

Table S1. Antibiotic susceptibility and resistance gene profiles for two *K. pneumoniae* clinical isolates.

Isolate name		*K.pneumoniae* FADDI-KP069		*K. pneumoniae* BM3
		Susceptibility	*MIC Value*	*Susceptibility*
Antibiotic susceptibility	Ampicillin	R	≥ 32	ND
	Amoxicillin/Clavulanic Acid	R	≥ 32	ND
	Ticarcillin/Clavulanic Acid	R	≥ 128	ND
	Piperacillin/Tazobactam	R	≥ 128	ND
	Cefazolin	R	≥ 64	ND
	Cefoxitin	R	≥ 64	ND
	Ceftazidime	R	≥ 64	ND
	Ceftriaxone	R	≥ 64	ND
	Cefepime	R	8	ND
	Meropenem	R	≥ 16	ND
	Amikacin	R	≥ 64	I
	Gentamicin	R	≥ 16	R
	Tobramycin	R	≥ 16	ND
	Ciprofloxacin	R	≥ 4	R
	Norfloxacin	R	≥ 16	ND
	Nitrofurantoin	R	256	S
	Trimethoprim	R	≥ 16	ND
	Trimethoprim/Sulfamethoxazole	R	≥ 320	ND
	Tetracyclin	ND	ND	R
	Chloramphenicol	ND	ND	S
	Netilmicin	ND	ND	I
	Tigecycline	ND	ND	I
	Fosfomycin	ND	ND	S

	Aztreonam	ND	ND	R
	ESBL	+		ND
	Carbapenemase (metallo or KPC)	+		ND
Resistance genes	NDM-1	ND		+
	CTX-M	ND		+
	CMY-2	ND		−
	SHV	ND		+
	TEM	ND		+
	AAC-6'-1B	+		+

R = Resistant; I = Intermediate; S = Susceptible.
